# The Use of Continuous Wavelet Transform Based on the Fast Fourier Transform in the Analysis of Multi-channel Electrogastrography Recordings

**DOI:** 10.1007/s10916-015-0358-4

**Published:** 2015-10-29

**Authors:** Dariusz Komorowski, Stanislaw Pietraszek

**Affiliations:** Faculty of Biomedical Engineering, Department of Biosensors and Biomedical Signals Processing, Silesian University of Technology, 40 Roosevelt’a street, 44-800 Zabrze, Poland; Institute of Electronics, Division of Biomedical Electronics, Silesian University of Technology, 16 Akademicka street, 44-100 Gliwice, Poland

**Keywords:** Electrogastrography, Continuous wavelet transform, Dominant frequency, Index of normogastria

## Abstract

This paper presents the analysis of multi-channel electrogastrographic (EGG) signals using the continuous wavelet transform based on the fast Fourier transform (CWTFT). The EGG analysis was based on the determination of the several signal parameters such as dominant frequency (DF), dominant power (DP) and index of normogastria (NI). The use of continuous wavelet transform (CWT) allows for better visible localization of the frequency components in the analyzed signals, than commonly used short-time Fourier transform (STFT). Such an analysis is possible by means of a variable width window, which corresponds to the scale time of observation (analysis). Wavelet analysis allows using long time windows when we need more precise low-frequency information, and shorter when we need high frequency information. Since the classic CWT transform requires considerable computing power and time, especially while applying it to the analysis of long signals, the authors used the CWT analysis based on the fast Fourier transform (FFT). The CWT was obtained using properties of the circular convolution to improve the speed of calculation. This method allows to obtain results for relatively long records of EGG in a fairly short time, much faster than using the classical methods based on running spectrum analysis (RSA). In this study authors indicate the possibility of a parametric analysis of EGG signals using continuous wavelet transform which is the completely new solution. The results obtained with the described method are shown in the example of an analysis of four-channel EGG recordings, performed for a non-caloric meal.

## Introduction

Electrogastrography is a research method designed for noninvasive assessment of gastric slow wave propagation [1–4]. One or multichannel EGG signals are obtained from the disposable electrodes, appropriately arranged on the surface of the abdomen of the patient’s stomach [5–7]. It is assumed that the frequency range of EGG signal is from 0.015 to 0.15 Hz and the amplitude of it is about 100–400 μV [6, 8]. The typical EGG examination takes about 2 h and consists of three parts: the first one - preprandial, usually no longer than 30 min, it is a stage before a meal (person under investigation should be fasted). The second part takes about 5 to 15 min, including time when the person accepts a standardized meal, and the third part - postprandial, about 60–120 min, when the meal is digested. The standard of a meal depends on the examining center. Most frequently three types of meals are used: non-caloric meal e.g., 400 ml water, liquid meal e.g., 250 ml of yogurt and the caloric meal, e.g., pancake with jam prepared according to a well-defined recipe [9, 10].

The initial analysis of EGG signals involves calculating dominant frequency and dominant power of slow waves [2, 4, 6, 11–13]. In the case of EGG examination the frequency is typically calculated in cycles per minute (cpm), as a medical standard [5]. According to the DF values, the EGG rhythm is classified to: bradygastria (*0.5–2.0 cpm*)*,* normal rhythm (*2.0–4.0 cpm*) or tachygastria (*4.0–9.0 cpm*) [4, 5]. Due to very high level of disturbances and interferences in EGG signals while receiving a meal, the DF values are calculated only for preprandial and postprandial parts. On the basis of the rhythm classification, the normogastria index is calculated [6]. This index is expressed as the amount of DF values in the range of normal rhythm to the total amount of the DF values [4, 5].

**Fig. 1:**
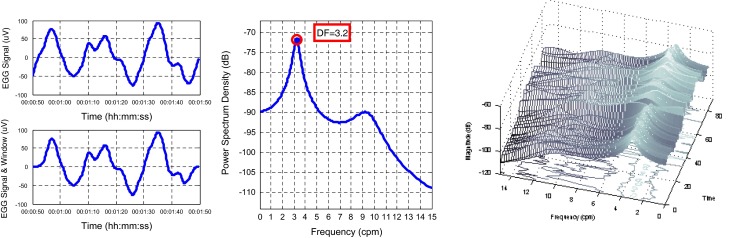
An example of 60s segment of EGG signal (*left*), its spectrum (*middle*) with marked DF and MP and an example of RSA analysis of one-channel EGG signal (*right*)

The parameters DF and MP are usually calculated by means of the spectral analysis. The spectral analysis of EGG is done for short segments (1 to 5 min length) of the signals. The values of DF and MP are calculated for each segment. The length of the segments depends on the limitations of the used method and is a compromise between accuracy and resolution of calculated frequency and its time location in the analyzed signal. Segments of 3 to 5 min length are used to calculate spectrum of the EGG signal, using the short-time Fourier transform (STFT), and for the nonparametric methods (for example AR, ARMA modeling), the minimum length of the segment is about 60 s [4, 6, 11, 12]. In the case of EGG signal analysis, the process of calculation the spectrum of consecutive or overlapped fragments is often referred as a running spectrum analysis (RSA) [8, 14]. Figure 1 shows a 1-min segment of the signal before and after application of Tukey window (left), its power spectrum (middle) with marked both DF and MP values, and an example of RSA analysis of one channel EGG signal (right).

The EGG signals analysis based on CWT is widely presented in the literature [15–19]. In this study, the CWT algorithm based on FFT was applied to determine the dominant frequency of the slow wave in the EGG signal. In the literature, this algorithm is referred to as CWTFT [20–22]. An application of CWT allows to reduce limitations of classical methods of spectrum analysis (e.g., FFT, STFT) and facilitates to determine instantaneous frequencies and its location in the time domain [23–25].

The continuous wavelet transform is a powerful tool for analyzing nonstationary time series signals in the time-frequency domain and substantially differs from the STFT method that allows clear localization on the time axis of the frequency components, existing in the analyzed signals. Such an analysis is possible using of variable width window which corresponds to the scale of observation (analysis). Wavelet analysis allows using long time windows when we need more precise low frequency information, and shorter when we need high frequency information. It should be noted that the wavelet analysis does not include the area of time-frequency (as in the case of STFT), but the area: the time-scale. The time-scale area can be converted to the appropriate area of pseudo-frequency - time, where the pseudo-frequency is the characteristic central frequency of the wavelet. Another important distinction from the STFT is that the CWT is not limited to sinusoidal analyzing function and do not requires, that the signal meets fairly strict criteria, which are required in the classical Fourier analysis [22].

## Method

The CWT reflects the correlation between the analyzed continuous-time signal *x*(*t*) and a function referred to as wavelets and is defined by the following formula

1 $$ Cw\left(a,b\right)={\displaystyle {\int}_{-\infty}^{+\infty }x(t){\psi_{a,b}}^{*}dt}=\frac{1}{\sqrt{a}}{\displaystyle {\int}_{-\infty}^{+\infty }x(t){\psi}^{*}\left(\frac{t-b}{a}\right)dt}, $$

where: *Cw*(*a*, *b*) is the function of the parameters *a* and *b*.

The *a* parameter is the dilation of wavelet (scale) and *b* defines a translation of the wavelet and indicates the time localization, ψ*(*t*) is the complex conjugate of the analyzing mother wavelet *ψ*(*t*) [22, 26]. The coefficient $\frac{1}{\sqrt{a}}$ is an energy normalized factor (the energy of the wavelet must be the same for different *a* value of the scale). Moreover, to be classified as a basic permissible wavelet, a wavelet function must satisfy the following mathematical criteria [27–30]:

The wavelet must have finite energy

2 $$ E={\displaystyle {\int}_{-\infty}^{+\infty }{\left|\psi (t)\right|}^2dt<\infty } $$

The following condition must hold true

3 $$ {C}_{\psi }={\displaystyle {\int}_{-\infty}^{+\infty}\frac{{\left|\overset{\wedge }{\psi}\left(\omega \right)\right|}^2}{\omega }d\omega <\infty }, $$

where

4 $$ \overset{\wedge }{\psi}\left(\omega \right)={\displaystyle {\int}_{-\infty}^{+\infty}\psi (t){e}^{-i\omega t}dt} $$

is the Fourier transform of the *ψ*(*t*) function and *ω* = 2*πf* is the circular frequency. This condition is defined as a condition of admissibility and can be interpreted as a requirement that |*ψ*(*ω*)|^2^ decay endeavored (head) to zero faster than $\frac{1}{\omega }$. This condition means that the wavelet has no zero frequency component, that is

5 $$ \overset{\wedge }{\psi }(0)=0 $$

Otherwise, the wavelet must have the zero mean value. In the literature the parameter *C*_*ψ*_ is called the admissibility constant. The value of *C*_*ψ*_ depends on the chosen wavelet [27–29].

In the case of complex wavelets, Fourier transform must have both a real component and a value of zero for negative frequencies [22].

As a result of CWT transform we obtain the two-dimensional function *E*(*a*, *b*) = |*Cw*(*a*, *b*)|^2^ called scalogram, which presents energy distribution of signals for used scales *a* and the time position *b* (locations). In practice, all functions that differ from |*Cw*(*a*, *b*)|^2^ only by the constant multiplicative factor are also called scalograms [22]. The CWT can be considered as a transform that converts the signal from the time domain to the scale-time domain. The scale as mentioned above can be converted to a frequency value (pseudo-frequency), the value of which depends on the center frequency of the applied wavelets and the scale value *a*

6 $$ {f}_a=\frac{f_c}{a}, $$

where: *f*_*a*_ is the frequency associated with the wavelet at the specific *a* scale, while *f*_*c*_ is the characteristic frequency of mother wavelet at scale *a = 1*, and time position *b = 0.* There is a very important distinction to be made here: “The characteristic frequency *f*_*c*_ of the wavelet used in the wavelet transform is representative of the whole frequency makeup of the wavelet. The wavelet does not contain a single frequency, and the signal is not decomposed according to numerous single (sinusoidal) frequencies; this is not Fourier analysis!” [22].

It is known, if the wavelet transform coefficients are given, it is possible to reconstruct the original signal by the inverse wavelet transform described by the following equation [20]

7 $$ x(t)=\frac{1}{K_{\psi }}{\displaystyle {\int}_{a=0}^{+\infty}\left({\displaystyle {\int}_{b=-\infty}^{+\infty }}Cw\left(a,b\right){\psi}_{a,b}(t)\frac{db}{a^2}\right)}da=\frac{1}{K_{\psi }}{\displaystyle {\int}_0^{+\infty }D}\left(a,t\right)da, $$

where *K*_*ψ*_ is a constant factor that depends on the applied wavelet function and the details function *D*(*a*, *t*) is given by the following equation

8 $$ D\left(a,t\right)={\displaystyle {\int}_{b=-\infty}^{+\infty}\frac{1}{a^2}Cw}\left(a,b\right){\psi}_{a,b}(t)db $$

In practical CWT applications usually discrete values of the *a* scale in the range of continuous values are used. As the result the wavelet coefficients are obtained (called wavelet series). The choice of appropriate wavelet function is crucial to obtain good results during signal analysis. The classic CWT transform is time consuming and it requires considerable computing power to apply it to the analysis of long signals.

In recent years, the new efficient algorithms have been developed for significant acceleration of CWT calculation. One of them uses well-known FFT algorithms to calculate the CWT [20, 22, 23].

If we define

9 $$ {\psi}_a(t)=\frac{1}{\sqrt{a}}\psi \left(\frac{t}{a}\right) $$

and

10 $$ {\psi}_{ab}(t)=\frac{1}{\sqrt[]{a}}\psi \left(\frac{t-b}{a}\right) $$

the definition of CWT transform (1) can be rewritten in the following form

11 $$ Cw\left(a,b\right)={\displaystyle {\int}_{-\infty}^{+\infty }x(t){\psi}_{ab}^{*}(t)dt}={\displaystyle {\int}_{-\infty}^{+\infty }x(t){\psi}_a^{*}\left(b-t\right)dt}, $$

which clearly indicates that the CWT can be treated as a convolution of the signal and wavelets. Consequently CWT can be expressed as an inverse Fourier transform

12 $$ Cw\left(a,b\right)=\frac{1}{2\pi }{\displaystyle {\int}_{-\infty}^{+\infty}\widehat{x}}\left(\omega \right){\widehat{\psi}}_{a,b}^{*}\left(\omega \right)d\omega, $$

where:

13 $$ {\widehat{\psi}}_{a,b}^{*}\left(\omega \right)=\sqrt{a}{\widehat{\psi}}^{*}\left(a\omega \right){e}^{i\omega b} $$

denote the Fourier transform of the analyzed wavelet at scale *a* and location *b* and

14 $$ \widehat{x}\left(\omega \right)={\displaystyle {\int}_{-\infty}^{+\infty }x(t){e}^{-i\omega t}dt} $$

is the Fourier transform of the analyzed signal *x(t).*

In the case of discrete signals (which are typical for the signals processing problems), assuming that the input signal x*(n)* includes *N* samples, the discrete versions of the convolution can be represented as

15 $$ {W}_a(b)={\displaystyle {\sum}_{n=0}^{N-1}x(n){\psi}_a^{*}}\left(b-n\right) $$

We can easily notice that in order to obtain the CWT we have to calculate the convolution of the signal and wavelets for each value of the location *b* and repeat the calculations for each value of the scale *a.* In the case of two periodic sequences (signals) we can use the property described by Eq. (15) and apply fast algorithms for determining the discrete Fourier transform (DFT) to calculate the circular convolution [31]

16 $$ \begin{array}{cc}\hfill {\displaystyle {\sum}_{n=0}^{N-1}x(n){\psi}_a^{*}}\left(b-n\right)=\frac{1}{N}{\displaystyle {\sum}_{k=0}^{N-1}\widehat{x}(k){\widehat{\psi}}_a^{*}(k)}{e}^{i\frac{2\pi }{N} kb},\;\hfill & \hfill b=0,1,2\dots N-1\hfill \end{array} $$

where the discrete Fourier transform of *x*(*n*) signal is given by formula

17 $$ \begin{array}{cc}\hfill \widehat{x}(k)={\displaystyle {\sum}_{n=0}^{N-1}x(n){e}^{-i\frac{2\pi }{N}nk}},\hfill & \hfill k=0,1,2\dots N-1\hfill \end{array} $$

${\widehat{\psi}}_a$ is the discrete Fourier transform of the wavelet *ψ*_*a*_

18 $$ \begin{array}{cc}\hfill {\widehat{\psi}}_a(k)={\displaystyle {\sum}_{n=0}^{N-1}{\psi}_a(n){e}^{-i\frac{2\pi }{N}nk}},\hfill & \hfill k=0,1,2\dots N-1\hfill \end{array} $$

where *k* is an index of frequency.

Assuming that the signal is sampled at a frequency *f* = *f*_*s*_ , the sampling period is *Δt* = 1/*f*_*s*_ and in order to obtain the unit energy for each scale *a*, the wavelet function is normalized by the following formula [23]

19 $$ {\widehat{\psi}}_a\left(a{\omega}_k\right)=\sqrt{\frac{2\pi a}{\varDelta t}}\widehat{\psi}\left(a{\omega}_k\right), $$

where:

20 $$ {\omega}_k=\frac{2\pi k}{N\varDelta t} $$

The CWT can be expressed as the products of the inverse Fourier transform

21 $$ {W}_a(b)=\frac{1}{N}\sqrt{\frac{2\pi a}{\varDelta t}}{\displaystyle {\sum}_{k=0}^{N-1}\widehat{x}\left(\frac{2\pi }{N\varDelta t}k\right){\widehat{\psi}}^{*}\left(a\frac{2\pi }{N\varDelta t}k\right){e}^{i\frac{2\pi }{N} kb}} $$

The above described method was used for the EGG signals analysis. The calculations were made for various wavelets e.g.,: Morlet, Derivative of Gausian and Paul. During initial tests, authors have examined all available in the applied version of the Matlab (R2013b) types of wavelets for CWTFT algorithm: m-th order derivative of a Gaussian wavelet, analytic Morlet wavelet, non-analytic Morlet wavelet, non-analytic Morlet wavelet with zero mean, Mexican hat wavelet. The non-analytic Morlet wavelet was chosen because the obtained scalograms provided similar information consistent with the commonly known properties of EGG signals as the occurrence of the characteristic frequencies (e.g., 3 cpm) and typical changes in parameters after administration of the meal [3].

In our work all the presented results were obtained for the non-analytic Morlet wavelet with zero mean, specified in the Fourier transform domain by the following formula

22 $$ \widehat{\psi}\left(a\omega \right)=\frac{1}{\sqrt[4]{\pi }}\left({e}^{-\frac{{\left(a\omega -{\omega}_0\right)}^2}{2}}-{e}^{-\frac{\omega_0^2}{2}}\right), $$

where *ω*_0_ is the non-dimensional frequency parameter and its value was set to 6 and 15. If *ω*_0_ = 6 the admissibility condition is satisfied [23, 32].

The dependence of the pseudo-frequency scale on the chosen wavelet is given by

23 $$ {f}_a=\frac{1}{a\varDelta t\lambda}, $$

where *λ* is the Fourier wavelength (frequency Fourier factor) for the Morlet wavelet and it is given by the following formula [23]

24 $$ \lambda =\frac{4\pi a}{\omega_0+\sqrt{2+{\omega_0}^2}} $$

**Fig. 2:**
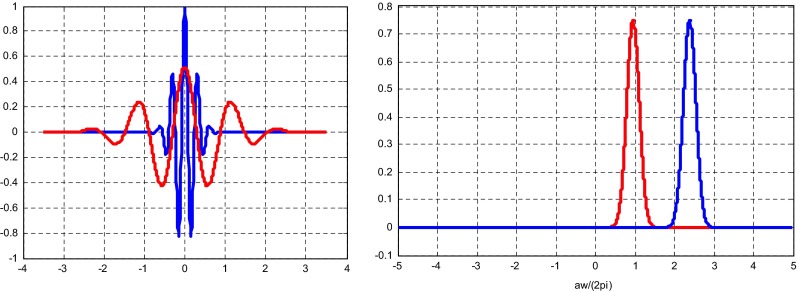
Morlet wavelet (*blue a* = 1, *red a* = 3.75) in the time domain (*left*) and in the frequency domain(*red* ω_0_ = 6, *blue* ω_0_ = 15) (*right*)

During calculations the range of the *a*_0_ = *ω*_0_*Δt* scale (1.5 and 3.75) to 50 in steps of 0.15 were used, which correspond to the range of the pseudo-frequency values 0.6453–0.0040 Hz for *ω*_0_ = 6 and 0.638–0.0039 Hz for *ω*_0_ = 15 , respectively. The examples of the applied Morlet wavelet are shown in the Fig. 2.

**Fig. 3:**
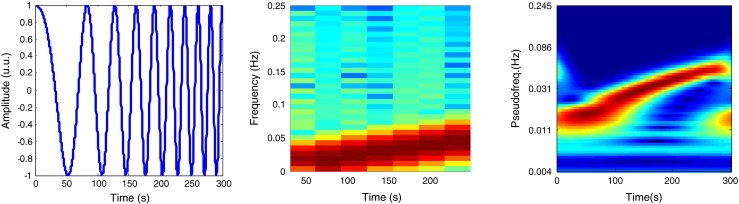
Segment of a chirp signal (*left*) associated with STFT transform (*medium*) and CWTFT (Morlet wavelet, ω_0_ = 6) transform (*right*)

The Fig. 3. depicts the deference between STFT and CWTFT transforms for an example of a chirp signal (from *0.005 to 0.025Hz*, sampled at *f*_*s*_ = 4*Hz*).

## Application to EGG

As the aim of this work is to show the application of new algorithms for EGG signal analysis, the paper presents the results of research carried out for only four subjects (women), volunteered to participate in the study. Their average age was 25.75 years (range: 24–31) and average BMI 19.83 (range: 18.6–21.1). Every volunteer gave a written consent to participate in the study. The research project was approved by the Bioethics Committee of the Silesian Medical University.

The duration of EGG study was in the range of 120 to 170 min and it consisted of three parts: preprandial (30–40 min), meal (5–10 min) and postprandial (90–120 min). Before the test, all participants were in a fasting state.

The calculations of the CWT coefficients (absolute values of elements *W*_*ab*_ further denoted |*W*_*ab*_| ) were performed for four-channels EGG signals, sampled at the frequency 4 Hz and the resolution of 12 bits, using the 4-channel prototype biomedical amplifier with the input range *±1 mV* and gain *5000*.

In order to assess the accuracy of the obtained results, the values of normogastria index (NI) were calculated for each EGG channel using the following procedure. The signal was divided into 60 s length segments (with overlap 50 %) and the |*W*_*ab*_| matrices were calculated. The |*W*_*ab*_| matrices were reshaped in order to remove the components which corresponded to the pseudo-frequency greater than 9 cpm (0.15Hz), because the analysis of EGG signals above this frequency is not carried out [2, 4, 5]. Then, for each segment of the modified |*W*_*ab*_| matrices the maximum value which corresponds to the pseudo-maximum power *pMP*(*l*) was found and its corresponding value of the dominant pseudo-frequency *pDF*(*l*), where *l* is the number of 1-min segment of the EGG signal. Finally *NI* index as the ratio of the number of *pDF* values in the range of 2–4 cpm to all values was calculated, for all channels of recorded EGG signal [2, 5, 6, 10]. Then, evaluated normogastria indexes were compared with those calculated by means of the classical method [4, 6].

## Results

**Fig. 4:**
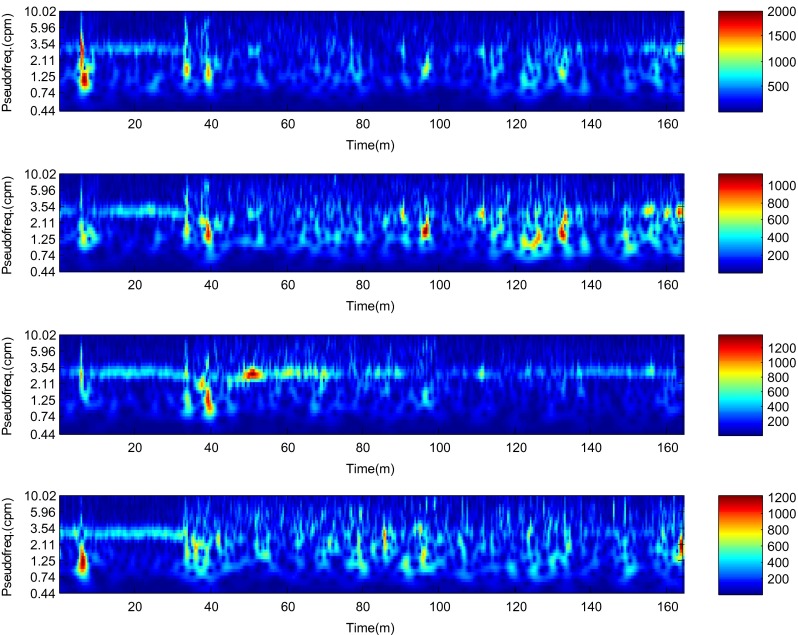
Time-frequency analysis with CWTFT (non-analytic Morlet wavelet, ω_0_ = 6) for four-channels EGG signal

**Fig. 5:**
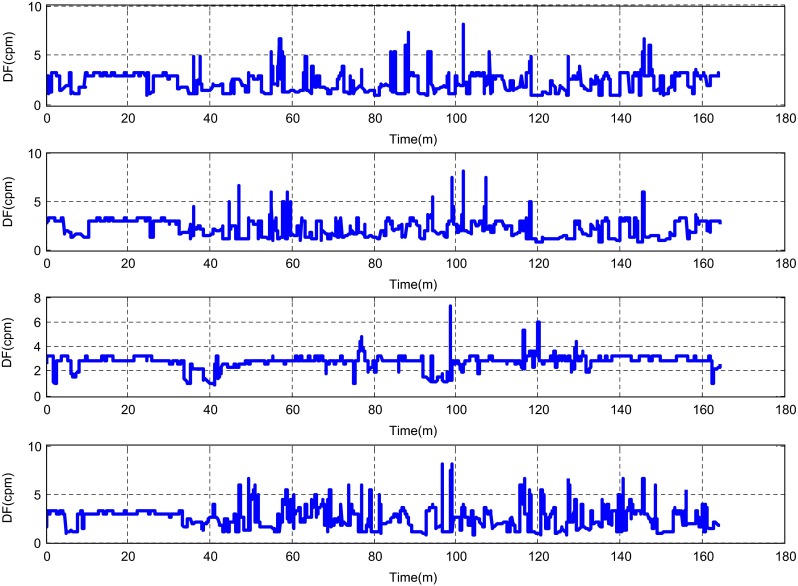
Instantaneous dominant frequency obtained by means of the CWTFT (non-analytic Morlet wavelet, ω_0_ = 6) analysis for four-channels EGG signal

**Fig. 6:**
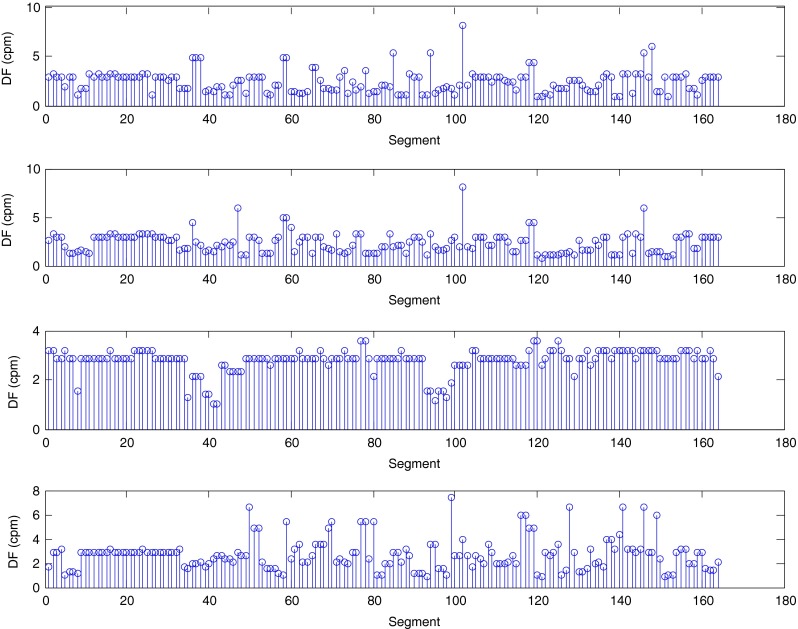
Dominant frequency (pDF) obtained by means of the CWTFT (non-analytic Morlet wavelet, ω_0_ = 6) analysis for four-channels EGG signal (for 60s segments)

**Fig. 7:**
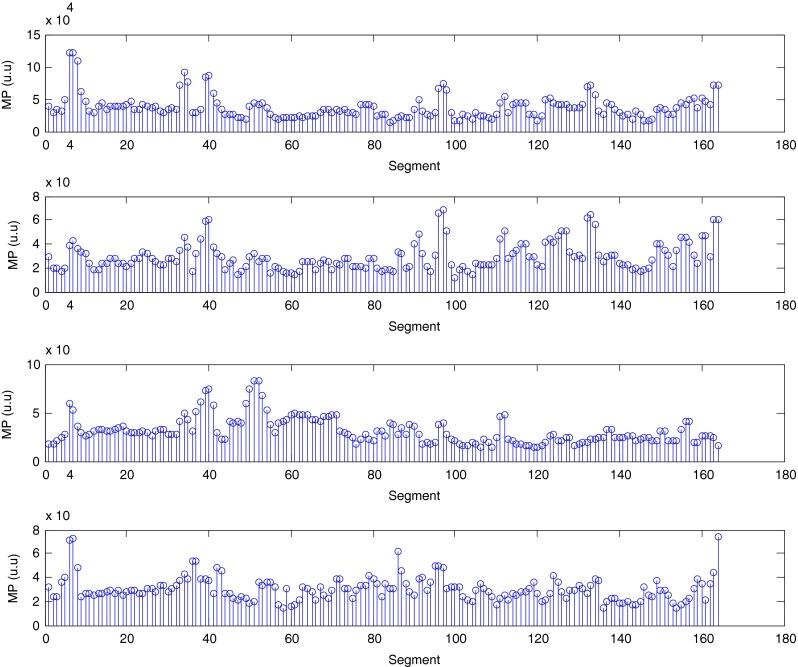
Maximum Power (pMF) obtained by means of the CWTFT (non-analytic Morlet wavelet, ω_0_ = 6) analysis for four-channels EGG signal (for 60s segments)

**Fig. 8:**
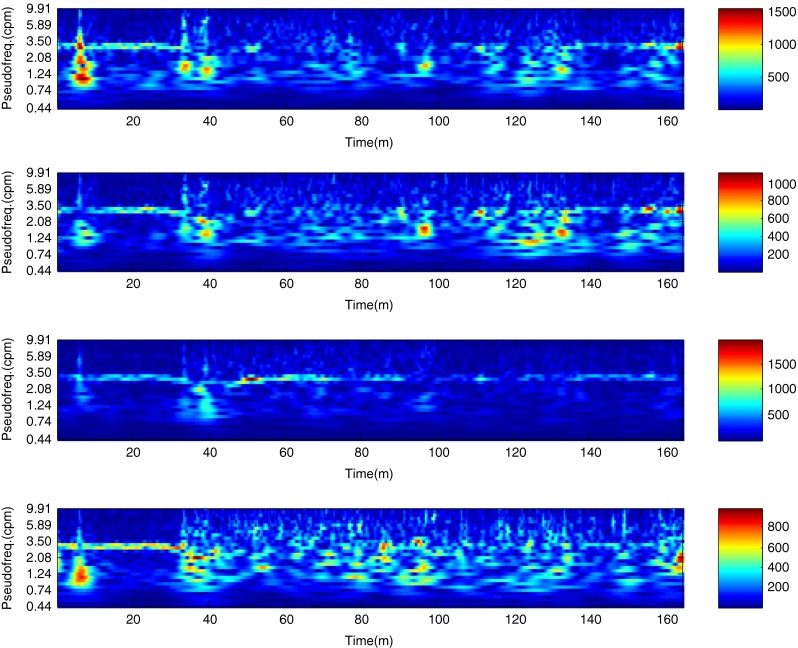
Time-frequency analysis with the CWTFT (non-analytic Morlet wavelet, ω_0_ = 15) for four-channels EGG signal

**Fig. 9:**
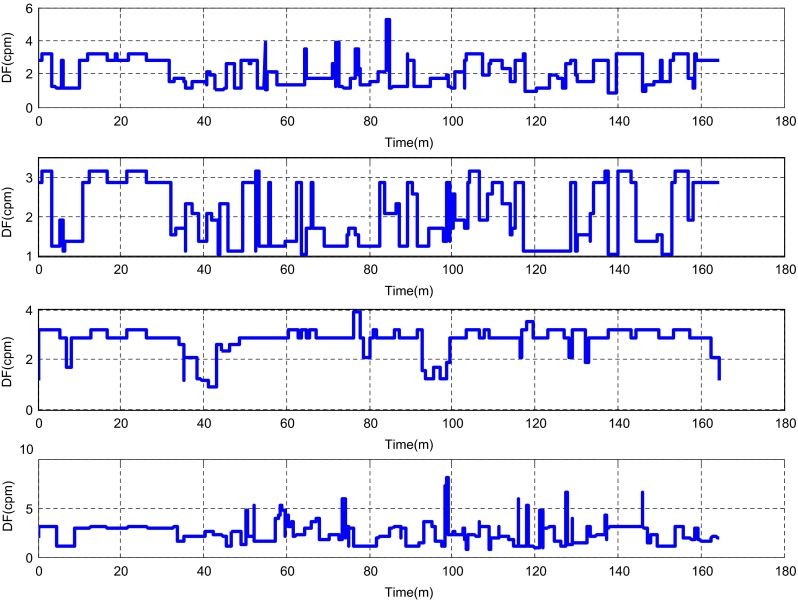
Instantaneous dominant frequency obtained by means of the CWTFT (non-analytic Morlet wavelet, ω_0_ = 15) analysis for four-channels EGG signal

**Fig. 10:**
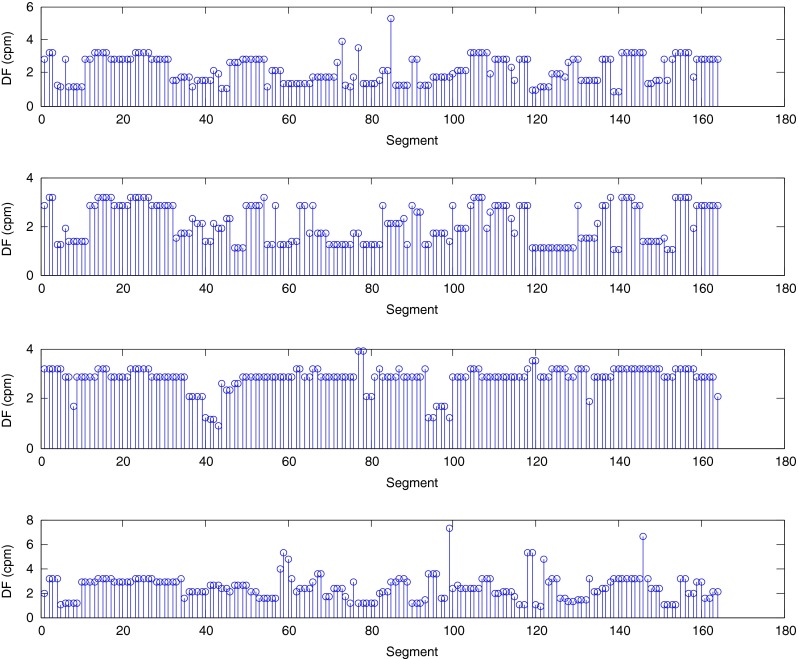
Dominant frequency (pDF) obtained by means of the CWTFT (non-analytic Morlet wavelet, ω_0_ = 15), analysis for four-channels EGG signal (for 60s segments)

**Fig. 11:**
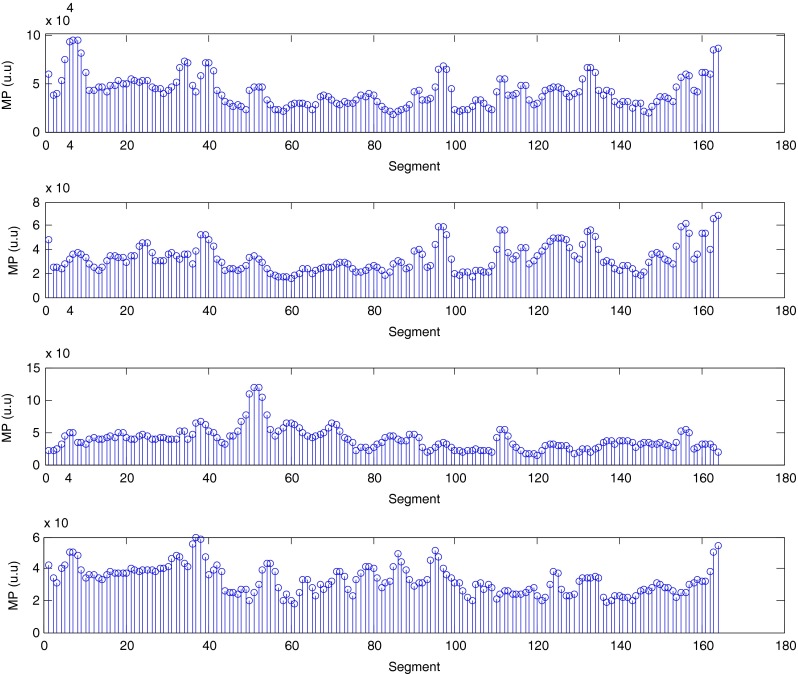
Maximum power (pMP) obtained by means of the CWTFT (non-analytic Morlet wavelet, ω_0_ = 15) analysis for four-channels EGG signal (for 60s segments)

**Fig. 12:**
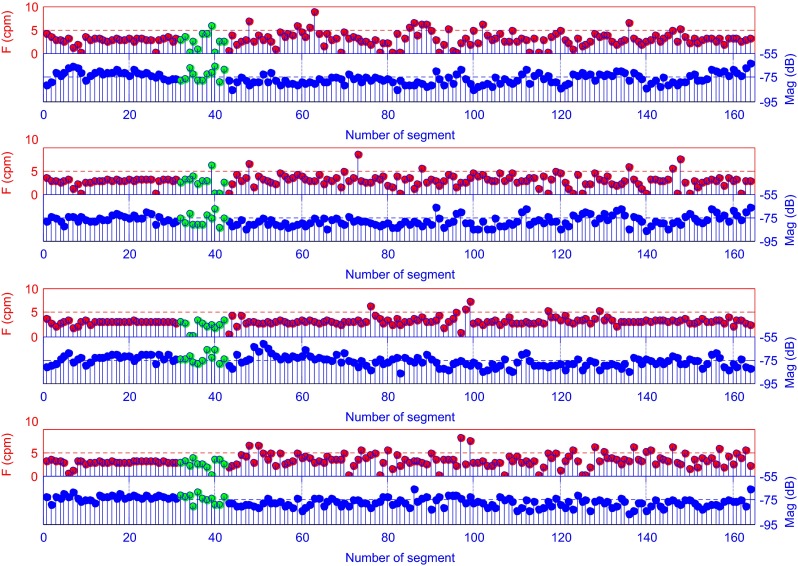
Dominant frequency (top) and MP (bottom) obtained by means of the classical method of EGG signal analysis (4-channels EGG signals, 60s segments) (the green color indicates the meal period)

The described method was applied to the analysis of over 2 h, four-channel EGG recordings, using a light non caloric liquid meal (400 ml cold water). Figure 4 shows the result of the CWT analysis using the non-analytic Morlet wavelet for ω_0_ = 6. The scale values were converted to the corresponding pseudo-frequency values. This figure clearly shows that the maximum value of the energy in the signal occurs about 3 cpm - the typical frequency of the slow wave in the EGG signal [4, 8]. Especially in the first part of the examination (preprandial) we can see clearly that the frequency is almost constant or oscillates near the 3 cpm. Figure 5 shows the frequency values corresponding to the maximum energy of scalogram. This process (curve) can be treated as a continuous frequency of the slow waves in the EGG signal. Figures 6 and 7 illustrate respectively the *pDF* and *pMP* for the sequent of 60 s segments of EGG signal. These values were also obtained based on the analysis of the CWTFT coefficients. Figures 6 and 7 were presented to compare the obtained results against the results of the classical EGG signals analysis (Fig. 12). The Figs. 8, 9, 10, and 11 show results of analysis using the Morlet wavelet with ω_0_ = 15.

The Fig. 12 shows the values of the DF and the MP for the same signal derived from the classical EGG analysis for the same EGG signals.

Normogastria indexes *Norm*_*I*_ and *Norm*_*60*_ (corresponding to NI index) were calculated by the proposed method while *Norm*_*c*_ was obtained by the means of classical method. These values of normogastria indexes for four EGG recordings are presented in Table 1 The normogastria indexes obtained on the basis of CWTFT were calculated by two methods: *Norm*_*I*_ was calculated on the basis of all the instantaneous frequency (corresponding to the maximum energy) and *Norm*_*60*_ was based on the average value for the 60 s segments.

**Table 1:** The comparison of normogastria indexes

Signal		CWT(FT) (Morlet, *ω*_0_ = 6 )		CWT(FT) (Morlet, *ω*_0_ = 15 )		Classical method
		*Norm*_*I*_	*Norm*_60_	*Norm*_*I*_	*Norm*_60_	*Norm*_*c*_
NS01A	ch1	0.549	0.594	0.589	0.616	0.752
	ch2	0.441	0.474	0.472	0.519	0.677
	ch3	0.224	0.286	0.190	0.210	0.534
	ch4	0.570	0.662	0.665	0.729	0.789
NS02A	ch1	0.819	0.854	0.908	0.915	0.939
	ch2	0.691	0.805	0.800	0.829	0.756
	ch3	0.791	0.866	0.803	0.817	0.829
	ch4	0.956	0.963	0.986	0.988	0.951
NS03A	ch1	0.740	0.754	0.779	0.821	0.873
	ch2	0.796	0.844	0.834	0.866	0.881
	ch3	0.845	0.858	0.883	0.873	0.896
	ch4	0.820	0.851	0.878	0.888	0.858
NS04A	ch1	0.257	0.307	0.289	0.331	0.551
	ch2	0.375	0.472	0.300	0.315	0.567
	ch3	0.908	0.937	0.971	0.976	0.921
	ch4	0.975	0.976	1.000	1.000	0.866

**Fig. 13:**
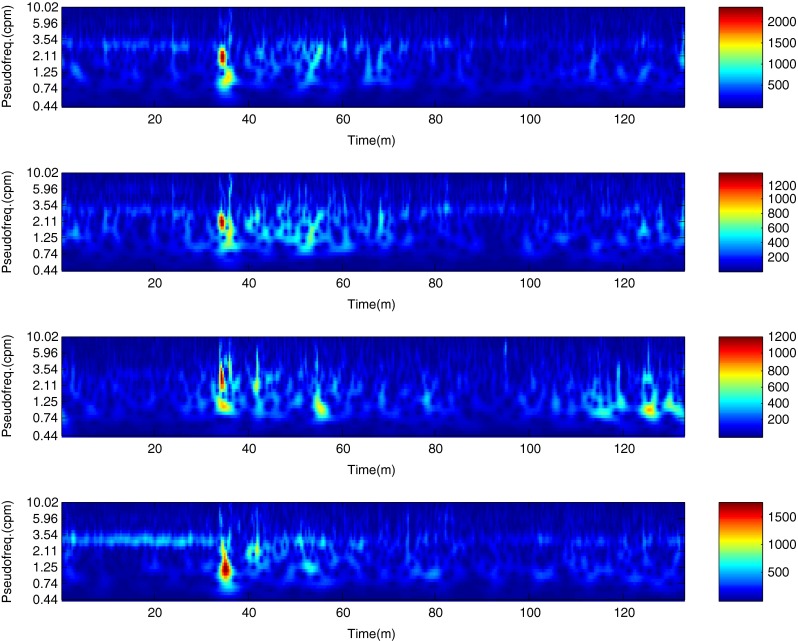
Time-frequency analysis with the CWTFT (non-analytic Morlet wavelet, ω_0_ = 6) for four-channels EGG signal

**Fig. 14:**
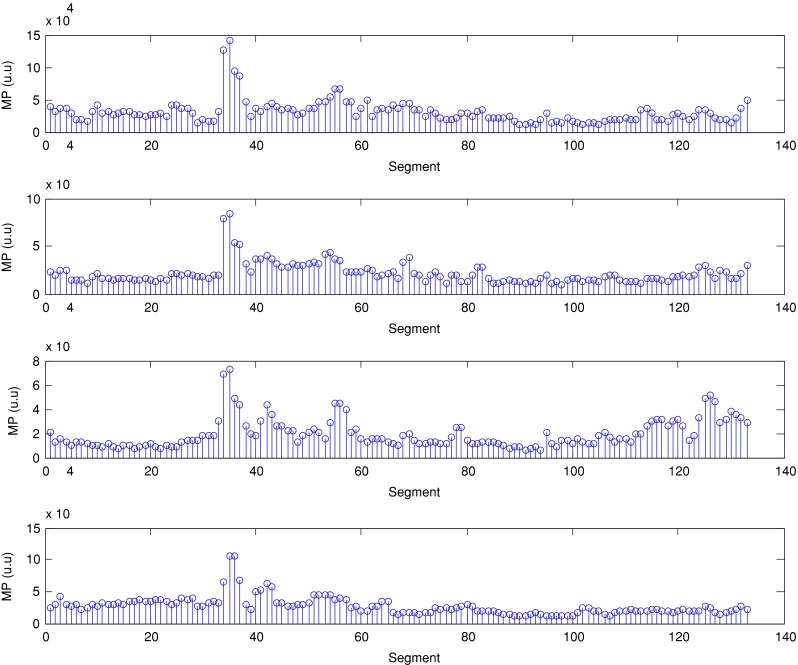
Maximum power obtained by means of the CWTFT (non-analytic Morlet wavelet, ω_0_ = 6) analysis for four-channels EGG signal (for 60s segments)

**Fig. 15:**
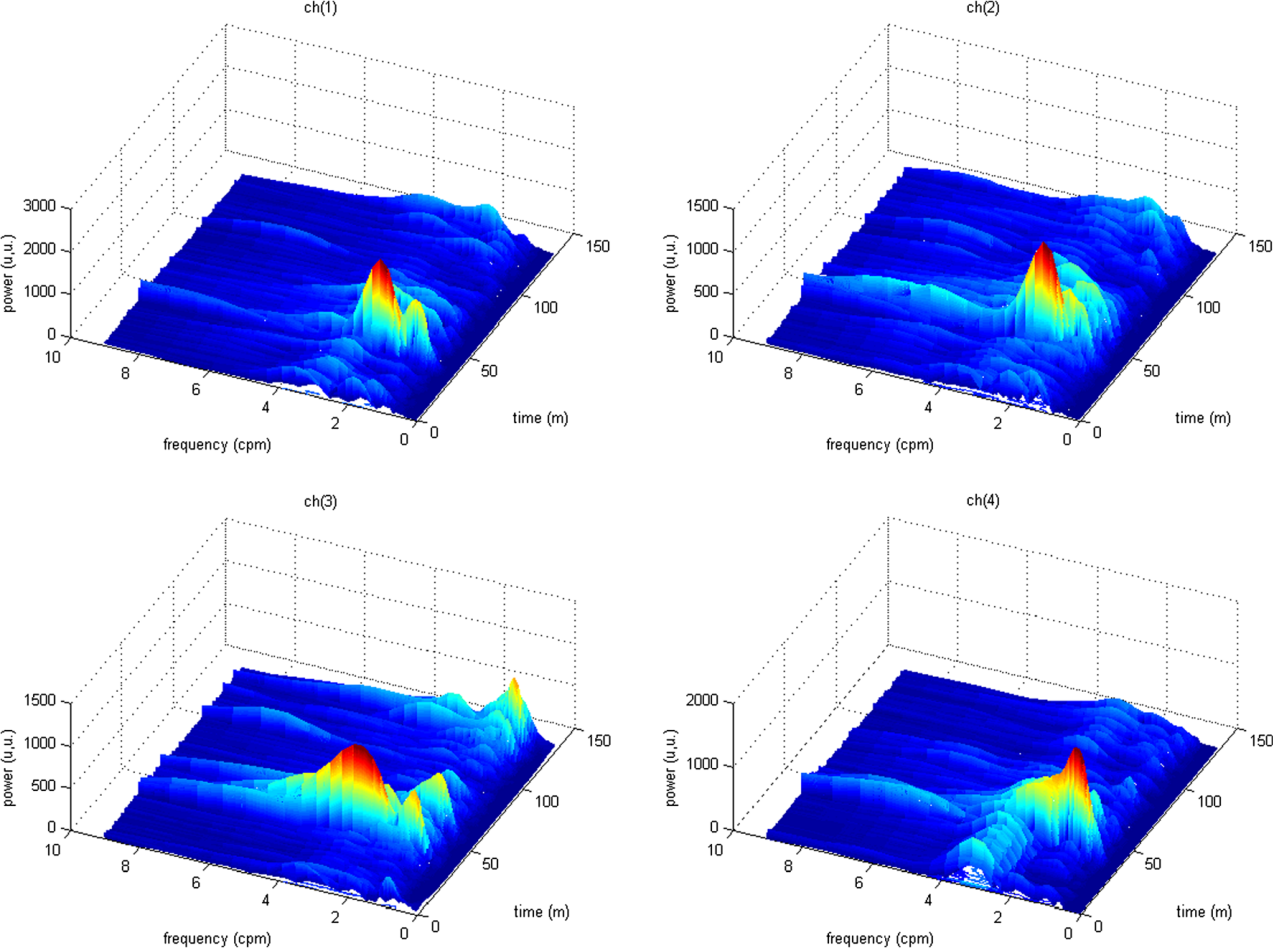
Pseudo-RSA (obtained by means of the CWTFT) of four-channels EGG signal

Figures 13, 14, and 15 show the results of the analysis for another EGG signal, which can clearly depict an immediate increase of power in the signal EGG after the meal and its gradual reduction during the postprandial phase. This is the typical phenomenon which can be often observed during the EGG examination [13].

## Conclusion

In this paper, the method of multi-channels EGG signals analysis by means of the CWTFT was described. Thanks to FFT applying in the CWT algorithm computation, the results for relatively long records of EGG can be obtained in a fairly short time (much faster than using the classical methods based on RSA analysis, e.g., using AR or ARMA models).

In this study, for the first time, the authors show the possibility of a parametric analysis of multi-channel EGG signals, using continuous wavelet transform. The normogastria indexes obtained by means of the proposed method, have similar values to the coefficients calculated by means of the classical analysis. However, it was noticed that the largest differences occurred in the signals for which the value of normogastria index was below 0.6. This phenomenon requires further investigation and necessary medical verification.

We can notice that results obtained by the analysis of the CWT are dependent on the mother wavelet function, which significantly affects the quality of signal analysis and results [16]. According to our tests, the Morlet wavelet gives the best results of the EGG signal analysis moreover preliminary results show the robustness of the method and its large potential in the future analysis of the EGG signals. Additionally, the presented method allows to determine the instantaneous values of the dominant frequency and maximum energy which was not possible with the classical EGG signal analysis.

The possibility of continuous observation of the dominant frequency and the dominant power (and other coefficients calculated on this basis) gives opportunities for a wider application of the proposed method in the medical diagnosis of digestive systems. From medical point of view, the described method must be clinically verified, which requires a sufficiently long time, adequate resources and a commitment of medical environments.
